# Family-to-Work Conflict and Innovative Work Behavior Among University Teachers: The Mediating Effect of Work Stress and the Moderating Effect of Gender

**DOI:** 10.3390/bs15101309

**Published:** 2025-09-25

**Authors:** Xiaohong Bao, Jia Dong, Jianwen Guo

**Affiliations:** 1 Special Education College, Changchun University, Changchun 130022, China; baoxh@ccu.edu.cn; 2 School of Educational Sciences, Nanjing Normal University, Nanjing 210023, China; dongjia0403@163.com; 3 College of Elementary Education, Capital Normal University, Beijing 100048, China

**Keywords:** family-to-work conflict, innovative work behavior, work stress, gender, Chinese university teachers

## Abstract

Family-to-work conflict is a common phenomenon among university teachers and can decrease their innovative work behavior. However, the mechanism underlying such conflict remains uncertain. To clarify the relationship between family-to-work conflict and innovative work behavior and explore the mechanism underlying this relationship, this study combines conservation of resources theory with gender role theory and employs the structural equation modeling (SEM) method. Questionnaires completed by 916 university teachers were analyzed with the assistance of SPSS 23.0 and AMOS 24.0. The findings reveal that family-to-work conflict negatively influences innovative work behavior and that challenge stress and hindrance stress significantly mediate the relationship between family-to-work conflict and innovative work behavior, albeit in opposite directions. Specifically, challenge stress exerts a positive mediating effect on the relationship between family-to-work conflict and innovative work behavior, whereas hindrance stress demonstrates a negative mediating effect in the association. Additionally, gender significantly moderates the relationships between family-to-work conflict and challenge stress and between family-to-work conflict and hindrance stress. These results shed light on the inherent mechanisms that govern the relationship between family-to-work conflict and innovative work behavior among university teachers and highlight the significance of two types of work stress, i.e., challenge stress and hindrance stress, as well as gender in this context. In addition, this research offers fresh insights that can support future investigations of how schools and governments can promote innovative work behavior among university teachers.

## 1. Introduction

In an increasingly competitive environment, innovation has become increasingly important (Song et al., 2023). In particular, innovation, including innovative work behavior, is crucial with respect to both the individual and the organization (Aryani et al., 2024). The claim also applies to teachers’ innovative teaching behaviors. Teachers’ innovative work behavior refers to innovative activities in which educators engage in the context of school teaching and learning. Specifically, this term refers to the processes by which teachers actively transform their teaching beliefs, proactively design and implement novel educational plans, and promote creativity and innovative performance among their students (M. Zhang & Zhang, 2012). Previous studies on this topic have reported that teachers’ innovative behavior can not only stimulate innovative thinking on the part of students but also promote progress in academic research, thus providing a continuous source of innovation that can facilitate social development (Y. Xu et al., 2024). Therefore, the task of enhancing teachers’ innovative work behavior is important (Song et al., 2023).

Previous research on this topic has identified several factors that influence teachers’ innovative behavior, such as the school organizational climate for innovation as well as teachers’ motivation to perform innovative work, educational qualifications, learning organization, work engagement, promotion focus and autonomous motivation; other relevant factors include family supportive supervisor behavior, teaching self-efficacy, and affective commitment (Aryani et al., 2024; Hosseini & Haghighi Shirazi, 2021; M. Li et al., 2016; Q. Li & Liu, 2023; M. Zhang & Zhang, 2012). Among these variables, family-to-work conflict, a significant challenge for teachers in the Chinese context, deserves particular attention. Studies have shown that family-to-work conflict negatively impacts teachers, leading to emotional exhaustion, reduced depersonalization, and diminished personal accomplishment (Tang, 2010). Moreover, although some studies have focused on the relationship between family-to-work conflict and teachers’ innovative work behavior (Solichin et al., 2023), the mechanisms underlying this relationship have received little attention. To address this issue, this study aims to clarify the relationship between teachers’ family-to-work conflict and innovative work behavior as well as to investigate the mechanisms underlying this relationship, particularly in light of the roles played by work stress and gender in this context.

The present study makes two innovative contributions to the literature on this topic. Theoretically, it pioneers the integration of Conservation of Resources (COR) theory with gender role theory to systematically examine the relationship between family-to-work conflict and innovative teaching behaviors among university teachers, specifically analyzing the mediating effects of challenge stress and hindrance stress while identifying gender as a moderator in the initial phase of the mediation pathway, thereby not only validating but also extending existing theoretical perspectives while enriching the knowledge base in this field. Practically, it establishes a comprehensive and nuanced framework that deepens understanding of the complex interplay among family-to-work conflict, work stress, gender, and innovative work behaviors, providing actionable insights for educational institutions and policymakers to implement targeted interventions—such as strengthening family support systems, mitigating family-to-work conflict, and alleviating work stress—ultimately enhancing teachers’ teaching innovation capabilities.

### 1.1. Family-to-Work Conflict and Teachers’ Innovative Work Behavior

In recent years, the conflict between teachers’ work and family roles has intensified as a result of rapid economic development and the continuous advancement of internet technology (Q. Li & Liu, 2023). Such conflict of two directional components is known as work-to-family or family-to-work conflict. The former term refers to situations in which work interferes with family life, whereas the latter refers to situations in which family life interferes with work (Netemeyer et al., 1996). This study focuses on the latter concept. The salience of family-to-work conflict among Chinese university teachers stems from the tripartite influence of work characteristics, role expectations, and family structural transformations. Firstly, the flexible scheduling inherent in academic professions (e.g., absence of fixed office hours) blurs the physical boundaries between work and domestic spheres, which consequently elevates family members’ expectations regarding their participation in household responsibilities. Secondly, as a highly educated demographic, university teachers typically exhibits intensified career aspirations, yet simultaneously faces multidimensional pressures including heightened research evaluation metrics, pedagogical innovation demands, and increasing administrative burdens. Lastly, China’s demographic transition characterized by low fertility rates, aging populations, and the two-child policy, family structures now manifest hybrid configurations of 4-2-1 and multi-child households. This dual imperative of elder care obligations and intensive parenting creates intergenerational support burdens and temporal constraints on child-rearing. The above three factors collectively shapes the distinctive family-to-work conflict paradigm in this professional cohort.

With respect to the relationship between family-to-work conflict and innovative work behavior, numerous researchers have reported consistent findings. For example, Ford (1996) suggested that workers who suffer from strain as a result of such conflicts are unlikely to engage in innovative behaviors. Furthermore, Song et al. (2023) reported that workers’ family-to-work conflict could significantly and negatively influence their innovative behavior. This finding is also applicable to teachers. For instance, Solichin et al. (2023) reported that work–family conflict negatively affects innovative teaching skills among Indonesian teachers; specifically, work-to-family conflict reduces these teachers’ use of innovative teaching practices.

Conservation of resources theory (Hobfoll, 1989) posits that people strive to retain, protect, and build resources and that the factors that threaten them refer to the potential or actual loss of these valued resources. According to this theory, family-to-work conflict, as a type of conflict that specifically reflects the influence of family affairs on work and the corresponding interference, directly depletes and affects an individual’s work-related state, thereby reducing resource-draining behaviors within the workplace. In light of previous research on this topic (X. Zhang & Bartol, 2010), it is evident that teachers’ innovative behavior can be resource-depleting. In other words, family-to-work conflict depletes university teachers’ psychological resources (e.g., attentional capacity), thereby reducing the cognitive resources available in the workplace and undermining the cognitive abilities required for innovation. Moreover, when family-to-work conflict intensifies, university teachers may adopt conservative strategies in professional settings, consequently diminishing their willingness to engage in innovative work behaviors as a risk-avoidance mechanism. Therefore, we propose the first hypothesis:

**H1.**  *Family-to-work conflict significantly negatively influences teachers’ innovative work behavior.*

### 1.2. Work Stress Mediates the Relationship Between Family-to-Work Conflict and Teachers’ Innovative Work Behavior

Among the many factors that influence this relationship, the impact of stress on individual innovative behavior cannot be ignored (Y. Xu et al., 2024). According to previous studies on this topic, work stress is a complex and broad concept that can encompass various types of stress, including those that exhibit fundamentally different or even opposing characteristics. Therefore, a more effective analysis of this concept requires a focus on specific dimensions. This situation implies that previous generalizations of the relationship between family-to-work conflict and work stress (such as Su & Jiang, 2023) as well as the relationship between work stress and innovative work behavior (such as Wu et al., 2024) should be approached with caution. Cavanaugh et al. (2000) divided work stress into challenge and hindrance-related self-reported stress and reported that work stress is differentially related to various attitudinal and behavioral work outcomes depending on the stressors that are evaluated in this context. This study similarly divides work stress into challenge-related self-reported stress and hindrance-related self-reported stress, as proposed by Cavanaugh et al. (2000). It subsequently explores the mediating roles played by the two types of stress in the relationship between family-to-work conflict and teachers’ innovative work behavior.

Challenge stress refers to self-reported work stress that is associated with challenging job demands (Cavanaugh et al., 2000). Previous studies have shown significant discrepancies regarding the relationship between family-to-work conflict and challenge stress. While some research indicated a pronounced negative correlation (Huang, 2024), others reported a significant positive association (Sun et al., 2021). However, considering the unique characteristics of university teachers—a highly educated population—this study posits that their comprehensive understanding of issues may lead to distinct stress-coping mechanisms. Specifically, when confronted with family-to-work conflict, they tend to adopt a positive perspective, transforming familial responsibilities such as child-rearing and elder care into motivational drivers for professional development. This cognitive reframing may consequently generate constructive pressure that fosters personal growth, career development, and achievement. This psychological mechanism aligns with the profound wisdom of traditional Chinese philosophy: “When Destiny is about to bestow a great mission upon an individual, it will first test their willpower, strain their physical endurance, deprive them of sustenance, and leave them in hardship. It will disrupt their endeavors to temper their spirit, strengthen their character, and ultimately cultivate their latent potential.”

With respect to the relationship between challenge stress and innovative work behavior, some previous studies have reported that challenge stress exerts a significantly positive influence on innovative behavior (Du et al., 2014; Xie et al., 2024). This finding can be attributed to the inherent characteristics of challenge stress itself. Specifically, challenge stress may be related to positive feelings (e.g., eustress or challenge) (Cavanaugh et al., 2000), which can encourage individuals to confront and overcome obstacles, thereby fostering both personal and professional growth. Similarly, challenge stress should be associated with high motivation because people are likely to believe that there is a positive relationship between effort expended on coping with these demands and the likelihood of meeting the demands and also likely to believe that if these demands are met, valued outcomes will occur (Lepine et al., 2005). From this perspective, teachers are more inclined to invest effort in coping with challenge stress in order to achieve outstanding professional performance. Moreover, according to conservation of resources theory, when individuals experience challenge stress, they tend to engage in their work more proactively and seek to engage in innovative behaviors with the goal of acquiring additional resources. Therefore, we propose the second hypothesis:

**H2.**  *Challenge stress mediates the relationship between family-to-work conflict and innovative work behavior.*

Hindrance stress refers to stress that is associated with job demands or work circumstances that involve excessive or undesirable constraints that can interfere with or hinder an individual’s ability to achieve valued goals (i.e., demands that produce distress) (Cavanaugh et al., 2000). Regarding the relationship between family-to-work conflict and hindrance stress, recent research has reported that work–family conflict has a positive significant effect on hindrance stress (Freire & Bettencourt, 2022). From an emotional perspective, some studies have reported that family-to-work conflict is significantly closely related to depression and anxiety or that family-to-work conflict negatively impacts depression (Luo, 2025; Y. Wang & Peng, 2017). Since depression and anxiety are different types of negative feelings, the latter constitute an emotional characteristic of hindrance stress (Cavanaugh et al., 2000). Accordingly, we argue that the positive relationship between family-to-work conflict and hindrance stress has been validated in further detail.

With respect to the relationship between hindrance stress and innovative work behavior, some researchers have claimed that hindrance stress exerts a negative impact on innovative behavior (Du et al., 2014). Hindrance stress has generally been viewed as a negative form of stress that is characterized by obstacles and difficulties that can hinder an individual’s ability to achieve work-related goals. This type of stress can elicit feelings of pressure and frustration and may suppress individuals’ innovative behavior and career development (T. Xu et al., 2024). Conservation of resources theory also supports this relationship. According to this theory, when individuals encounter hindrance stress, they experience negative feelings such as distress and frustration (Cavanaugh et al., 2000). In such situations, people commonly perceive stress as a potential threat to personal growth and well-being, which leads them to adopt a defensive resource conservation strategy. This manifests in a tendency to rely on routine problem-solving approaches and exhibit diminished innovative behaviors (Du et al., 2014). Therefore, we propose the third hypothesis:

**H3.**  *Hindrance stress mediates the relationship between family-to-work conflict and innovative work behavior.*

### 1.3. Gender Moderates the Relationship Between Family-to-Work Conflict and Work Stress

Although the previous sections featured a review of the literature on the relationships between family-to-work conflict and both challenge stress and hindrance stress and proposed relevant hypotheses, importantly, the nature of this relationship may also differ across genders. The patriarchal system in Chinese traditional agrarian societies not only engendered and perpetuated hierarchical gender relations favoring men over women but also institutionalized the domestic gender division of labor characterized by men’s dominance in external affairs and women’s confinement to household responsibilities (Wei & Sang, 2019). The concept has given rise to numerous culturally resonant expressions, such as the Chinese proverb “A man is a rake, a woman is a box”, which metaphorically encapsulates the traditional gender division of labor in agrarian societies. Specifically, men have primarily been responsible for earning income and covering family expenses, whereas women have taken charge of domestic duties such as caring for children and elderly persons, cooking, and maintaining the household. However, as a result of social transformation, economic development, and increasing awareness among women, this rigid division of labor has gradually been dismantled. Some men now ascribe increasing importance to family roles in addition to their traditional work roles, and some women attribute increasing importance to their work roles in addition to their traditional family roles (Cinamon & Rich, 2002). In this context, this paper argues that both men and women now experience family-to-work conflict. However, in the context of family-to-work conflict, men and women experience different levels of work-related stress.

From the perspective of gender role expectations, men have typically been expected to fit the work profile most frequently and the family profile least frequently; i.e., they have tended to attribute high levels of importance to their work role and relatively low levels of importance to their family role. Specifically, Chinese society also places greater expectations on men to achieve success in their careers; thus, men’s occupational goals and aspirations frequently exceed those of women (Cinamon & Rich, 2002). Under this cultural framework, family-to-work conflict may manifest with lower intensity among male populations. Moreover, longstanding beliefs—for example, “married men with children are more career-driven”—have highlighted the positive influence of family on work. Responsibilities such as caring for elderly parents, raising children, and providing a better life for one’s family serve as motivating factors that encourage men to work more diligently. In this context, family does not constitute a negative factor for men; rather, it functions as a motivational force that encourages them to invest greater time and effort into their work, while simultaneously increasing their willingness to accept challenges and adopt a proactive stance in confronting and overcoming these difficulties. Thus, among men, the positive impact of family-to-work conflict on challenge stress is more pronounced. However, more women than men fit the family profile, which focuses on individuals who attribute high levels of importance to the family and relatively low levels of importance to work. Thus, among women, work that interferes with family obligations is viewed as less appropriate or tolerable and is thus more likely to be treated as a form of conflict. In contrast, when family, which is the most important domain for many women, interferes with work, this situation may be troublesome, but it is viewed as natural and expected, and women may not even interpret this kind of discord as a form of conflict (Cinamon & Rich, 2002). This suggests that women not only experience lower levels of family-to-work conflict, but they are also not expected to adopt measures such as reducing family time, increasing work hours, or developing work-related skills to resolve such conflicts. Consequently, they face lower levels of challenging work stress. Therefore, we propose the fourth hypothesis:

**H4.**  *Gender moderates the relationship between family-to-work conflict and challenge stress. Specifically, this relationship is stronger among men but weaker among women.*

On the basis of the preceding discussion regarding the roles and positions of men and women in both the family and professional spheres, it can be inferred that the roles that men play also imply that in situations involving family-to-work conflict, their family members are likely to provide them with additional support to help them overcome such conflict, thus mitigating the negative impacts of family-to-work conflict. However, concurrent with this dynamic, China’s socio-cultural expectations positioning men as primary economic providers necessitate their competence in meeting occupational demands, employer expectations, and maintaining clear career trajectories with superior job performance. Stated differently, within the framework of gendered role expectations, family-to-work conflict shows a stronger impact on hindrance stress among male employees.

In contrast, the increased pressure of social life and the escalating demands of work and family on Chinese professional women have given rise to a need for women to develop themselves and excel in the same manner as their male counterparts in the workplace. However, social achievement and family responsibility have doubled the load faced by professional women as well as the role conflict between their families and careers (Y. Wang & Peng, 2017). In situations involving family-to-work conflict, societal norms often primarily emphasize women’s responsibilities to manage their families duties, particularly those pertaining to motherhood, thus leading to inadequate support for their professional roles. Moreover, in contemporary society, children have ascended to the central position within families, with accompanying and nurturing them emerging as parents’ primary responsibility and a new ethical imperative for maintaining familial intimacy. As mothers bear the majority of caregiving duties in family education (Chang & Hu, 2022), they have already invested significantly more time and effort into their children. Given that individuals’ time and energy are finite, the substantial investment of women in the family—particularly in child-rearing—necessitates lower expectations placed upon them in the workplace. This suggests that women are more likely to accept, confront, and overcome negative workplace factors, such as ambiguous career prospects. Consequently, family-to-work conflict exerts a relatively weaker influence on hindrance stress among women. We thus propose the fifth hypothesis:

**H5.**  *Gender moderates the relationship between family-to-work conflict and hindrance stress. Specifically, this relationship is stronger among men but weaker among women.*

### 1.4. Aims of the Present Study

**Figure 1:**
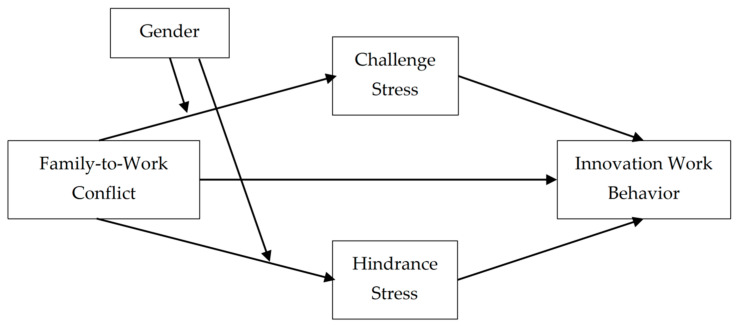
The moderated mediation model tested in this research.

Figure 1 illustrates the framework underlying this study, which primarily investigates the impact of family-to-work conflict on innovative behavior among university teachers. This research focuses on the mediating effects of challenge stress and hindrance stress on this relationship as well as the moderating effect of gender.

## 2. Method

### 2.1. Participants and Procedure

A total of 1100 full-time university teachers (with no restrictions on institutional type or academic discipline), in the central region of mainland China, were recruited and completed a set of questionnaires that included the family-to-work conflict subscale, the work stress scale and the innovative work behavior among a questionnaire for teachers. A total of 184 questionnaires (16.73%) were excluded from subsequent analyses as a result of poor response quality (e.g., patterned responses, cyclical response patterns, extreme response bias). The final sample consisted of 916 participants (Mage = 36.85, SD = 7.84). The detailed demographic characteristics of the participants are presented in Table 1.

An online survey using a non-probability sampling design was conducted between 1 April and 1 June 2024, with data collected via the Wenjuanxing platform. At the beginning of the questionnaire, the participants were informed about the content of the survey as well as their rights. This information included the voluntary nature of their participation, their ability to withdraw from this research at any time, and an assurance that their responses would remain strictly confidential. All the methods used in this study were in line with the principles of the Declaration of Helsinki.

### 2.2. Measures

#### 2.2.1. Family-to-Work Conflict

Family-to-work conflict was measured via the 4-item Family Interference with Work Conflict subscale of the Work–Family Conflict scale (WFC; Carlson et al., 2000; Liu et al., 2017). Sample items included “As a result of my numerous family responsibilities, I often feel exhausted at work”; “Pressure from my family frequently causes me to think about personal matters at work”; “To prioritize my family, I tend to relax my focus on work”; and “Pressure from my family has a significant impact on my work performance”. The items included in this measure were scored on a 5-point scale ranging from 1 (extremely disagree) to 5 (extremely agree); higher average scores on these four items indicated higher levels of family-to-work conflict. A confirmatory factor analysis (CFA) revealed that the scale exhibited a good fit to the data (*χ*^2^/*df* = 5.90, RMSEA = 0.07, NFI = 1.00, TLI = 0.98, GFI = 1.00, IFI = 1.00, CFI = 1.00, SRMR = 0.01), and the 90% confidence interval for RMSEA was [0.03, 0.14]. The Cronbach’s alpha coefficient of this measure in the present study was 0.83.

#### 2.2.2. Work Stress

Work stress was measured via the 11-item Work Stress Scale developed by Cavanaugh et al. (2000). This questionnaire included two dimensions, i.e., challenge stress (e.g., “Time pressures that I experience”) and hindrance stress (e.g., “The amount of red tape with which I must deal to perform my job”), which were associated with 6 and 5 items, respectively. The items included in this measure were scored on a 5-point scale ranging from 1 (extremely disagree) to 5 (extremely agree); higher average scores indicated higher levels of work stress. For the purpose of enhancing the overall validity of the model, we excluded an item (“The amount of red tape I need to go through to get my job done”) with suboptimal factor loading during the scale refinement process. A CFA revealed that the final version of scale exhibited a good fit to the data (*χ*^2^/*df* = 5.94, RMSEA = 0.07, NFI = 0.96, TLI = 0.96, GFI = 0.96, IFI = 0.97, CFI = 0.97, SRMR = 0.06), and the 90% confidence interval for RMSEA was [0.06, 0.08]. The Cronbach’s alpha coefficients of the two dimensions and the total questionnaire were 0.92, 0.80, and 0.86, respectively.

#### 2.2.3. Teachers’ Innovative Work Behavior

Teachers’ innovative work behavior was measured via the 16-item Teacher Innovative Work Behavior Questionnaire (TIWBQ) developed by M. Zhang and Zhang (2012). This questionnaire included three dimensions, i.e., innovative ideas (e.g., “I actively seek to learn from the teaching experiences of others”), innovative actions (e.g., “I employ a diverse range of instructional methods to impart both knowledge and skills effectively”), and innovative results (e.g., “I encourage my students to engage critically with and discuss controversial issues”), which were associated with 4, 6, and 6 items, respectively. The items included in this measure were scored on a 5-point scale ranging from 1 (extremely noncompliant) to 5 (extremely compliant); higher average scores indicated higher levels of innovative work behavior among teachers. A CFA revealed that the scale exhibited a good fit to the data (*χ*^2^/*df* = 3.58, RMSEA = 0.05, NFI = 0.94, TLI = 0.95, GFI = 0.95, IFI = 0.96, CFI = 0.96, SRMR = 0.04), and the 90% confidence interval for RMSEA was [0.05, 0.06]. The Cronbach’s alpha coefficients of the three dimensions and the total questionnaire were 0.80, 0.85, 0.84, and 0.90, respectively.

#### 2.2.4. Gender

The participants reported their gender by responding to the following item: “Please report your gender”. Two genders were included in this research: male and female.

### 2.3. Method of Data Analysis

Firstly, we used SPSS 23.0 software to investigate common method bias, multicollinearity, and descriptive statistics in this context; we then used Pearson’s correlation to assess the variables of interest. Secondly, we use structural equation modeling (SEM) with the assistance of AMOS 24.0 software with the aim of analyzing the moderated mediation model hypothesized as part of the current study. The significance of the indirect and moderated mediating effects was tested by setting the number of bootstrapped resamples to 5000 and the bias-corrected confidence interval to 95%. The 95% bias-corrected confidence intervals did not contain zero, thus indicating that the indirect and moderated mediating effects were significant (Preacher & Hayes, 2008). Additionally, a multigroup SEM analysis was conducted to determine whether the proposed model would vary statistically between male and female university teachers for each path coefficient.

## 3. Results

### 3.1. Preliminary Analysis

Harman’s single-factor test was performed to detect the possible presence of common method bias in our research (Podsakoff et al., 2003). The findings revealed a multifactor structure; the highest loading factor accounted for 23.33% of the total variance, i.e., less than 40%, thus indicating that common method bias was not significant in this study. In addition, the variance inflation factor (VIF) values ranged from 1.51 to 3.23, i.e., less than the threshold value of 10 (Kline, 2015), thus indicating that multicollinearity was not significant in this study.

The means, standard deviations and intercorrelations among the variables included in this research are presented in Table 2. Family-to-work conflict was significantly and positively related to both challenge stress (r = 0.20, *p* < 0.001) and hindrance stress (r = 0.40, *p* < 0.001), and challenge stress was significantly and positively related to both hindrance stress (r = 0.30, *p* < 0.001) and innovative work behavior (r = 0.09, *p* < 0.01). In addition, both family-to-work conflict (r = −0.18, *p* < 0.001) and hindrance stress (r = −0.25, *p* < 0.001) were significantly and negatively related to innovative work behavior.

The CR of family-to-work conflict, challenge stress, hindrance stress, innovative work behavior were 0.82, 0.92, 0.80 and 0.95, respectively, denoting high composite reliability based on the principle of Hair et al. (1998). The AVE of these scales were 0.54, 0.66, 0.51, and 0.51, and the square root of the AVE on the diagonal of the scale was higher than the correlation among these scales, indicating that these questionnaire have strong convergent validity and discriminant validity based on the principle of Fornell and Larcker (1981). Addition, the HTMT between family-to-work conflict, challenge stress, hindrance stress, innovative work behavior were less than the threshold value of 0.85, further denoting high composite reliability based on the principle of Henseler et al. (2015). The detailed statistics are presented in Table 2.

### 3.2. The Mediating Effects of Challenge Stress and Hindrance Stress

**Figure 2:**
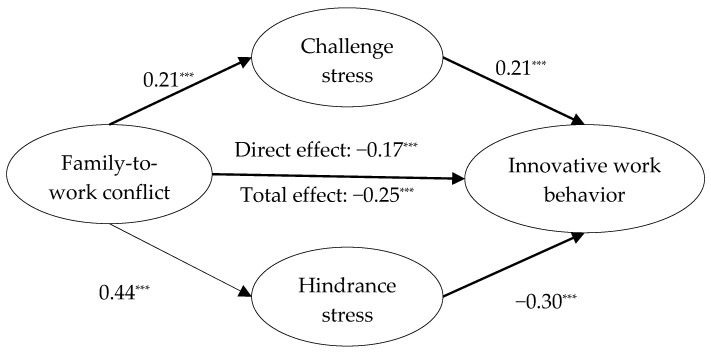
Standardized path coefficients in the structural model (MGSEM). Note: *** *p* < 0.001.

The SEM depicted in Figure 2 was constructed with family-to-work conflict as the independent variable, challenge stress and hindrance stress as mediating variables, innovative work behavior as the dependent variable, while controlling for age, education background, teaching experience and professional titles. The mediation model exhibited a good fit to the data (*χ*^2^/*df* = 3.86, CFI = 0.90, TLI = 0.90, RMSEA = 0.06, SRMR = 0.07). As illustrated in Figure 2, the total and direct effects of family-to-work conflict on innovative work behavior (β = −0.25, *SE* = 0.04, 95% confidence interval (CI) = [−0.34, −0.16], *p* < 0.001; β = −0.17, *SE* = 0.05, 95% CI = [−0.26, −0.07], *p* < 0.001) were significant; thus, H1 was supported. In addition, family-to-work conflict significantly and positively predicted challenge stress (β = 0.21, *SE* = 0.04, 95% CI = [0.13, 0.29], *p* < 0.001), and challenge stress significantly and positively predicted innovative work behavior (β = 0.21, *SE* = 0.05, 95% CI = [0.12, 0.31], *p* < 0.001). The 95% confidence intervals pertaining to the indirect effect of family-to-work conflict on innovative work behavior [0.01, 0.03] via challenge stress did not include zero, thus confirming the presence of a significant mediating relationship in this context. In summary, H2 was supported.

Family-to-work conflict significantly and positively predicted hindrance stress (β = 0.44, *SE* = 0.04, 95% CI = [0.36, 0.52], *p* < 0.001), and hindrance stress significantly and negatively predicted innovative work behavior (β = −0.30, *SE* = 0.05, 95% CI = [−0.39, −0.20], *p* < 0.001). The 95% confidence intervals pertaining to the indirect effect of family-to-work conflict on innovative work behavior [−0.07, −0.03] via hindrance stress did not include zero, thus confirming the presence of a significant mediating relationship. H3 was thus supported.

### 3.3. The Moderating Role of Gender in the Relationship Between Family-to-Work Conflict and Work Stress

A multigroup SEM analysis was conducted to determine whether all proposed paths varied across different gender conditions. The multigroup model indicated an adequate fit to the data: *χ*^2^/*df* = 2.53, RMSEA = 0.04, TLI = 0.89, IFI = 0.90, CFI = 0.90, SRMR = 0.08. Specifically, as shown in Table 3, the relationship between family-to-work conflict and challenge stress was significantly stronger among males (β = 0.35, SE = 0.09, 95% CI = [0.18, 0.51], *p* < 0.001) than among females (β = 0.16, SE = 0.05, 95% CI = [0.07, 0.24], *p* < 0.01). Critical Ratios (CR) indicated a significant difference (CR = 2.52, *p* < 0.05); thus, H4 was supported.

Furthermore, the relationship between family-to-work conflict and hindrance stress was significantly stronger among males (β = 0.62, SE = 0.07, 95% CI = [0.46, 0.75], *p* < 0.001) than among females (β = 0.37, SE = 0.05, 95% CI = [0.27, 0.46], *p* < 0.001). CR indicated a significant difference (CR = 2.87, *p* < 0.01); thus, H5 was supported.

## 4. Discussion

### 4.1. Family-to-Work Conflict and Teachers’ Innovative Work Behavior

The findings of this study, which were largely in line with the proposed hypotheses, indicate that family-to-work conflict has a negative and statistically significant effect on teachers’ innovative work behavior. These results are also in line with the outcomes reported by several previous studies, such as Z. Li and Yin (2021), Solichin et al. (2023), and Song et al. (2020).

These results can also be explained from the perspective of conservation of resources theory. According to the theory, individuals are more psychologically motivated when resources are available, and they proactively acquire such resources and combine them with the goal of generating greater value. Conversely, when individuals encounter threats pertaining to the loss of their own resources, they tend to behave in conservative ways with the aim of preserving their resources, thus leading them to avoid risky activities (Q. Li & Liu, 2023). When an individual’s family life, including caring for children, supporting elderly parents, fulfilling daily household responsibilities, and navigating family relationships, interferes with their work, fewer resources are available for work. In this study, innovative teaching behaviors were identified as complex; accordingly, such behaviors typically require teachers to invest substantial time and effort. The findings of this research thus suggest that teachers’ engagement in innovative work behaviors naturally decreases as a result of this situation.

### 4.2. The Mediating Role of Work Stress

Perhaps the most compelling findings of this research are those that identify both challenge stress and hindrance stress as mediators in the relationship between family-to-work conflict and innovative work behavior, albeit in opposite directions. These findings are in line with previous research on this topic; for example, Cavanaugh et al. (2000) identified challenge stress and hindrance stress as mediators with regard to the work outcomes in question, though in opposing directions.

Challenge stress mediates the relationship between family-to-work conflict and innovative work behavior; specifically, family-to-work conflict can improve innovative work behavior by improving challenge stress—a finding that is consistent with the results reported by Y. Xu et al. (2024). This study attributes this phenomenon to the following reasons. Firstly, university teachers typically exhibit high levels of education and cognitive ability, which allow them to adopt a more positive perspective on family-to-work conflicts. These teachers often perceive such conflicts as a form of challenging stress, thus motivating them to work more diligently with the aim of achieving higher levels of professional success. Secondly, when individuals perceive the stress that they face as a form of challenging work-related pressure, they are more likely to believe that such stress can lead to personal growth; thus, they tend to focus on the positive aspects of this stress (Dai et al., 2020), thereby not only increasing the difficulty of and resource consumption involved in their work but also eliciting a sense of accomplishment, thereby stimulating teachers’ innovative motivation (Y. Xu et al., 2024). Conservation of resources theory also posits that the challenge stress experienced by higher education teachers can promote innovative behavior by stimulating innovative motivation, facilitating resource integration, and driving practical exploration (Y. Xu et al., 2024). Hindrance stress mediates the relationship between family-to-work conflict and innovative work behavior; specifically, family-to-work conflict can decrease innovative work behavior by increasing hindrance stress, a conclusion that is in line with previous research conducted by Y. Xu et al. (2024). The underlying causes of this phenomenon may stem from individuals experiencing hindrance stress, which often leads to negative perceptions and pessimistic expectations. Such individuals frequently encounter feelings of oppression and frustration, ultimately impeding personal capability development, disrupting task completion, and hindering potential realization—consequently resulting in diminished innovative work behaviors (J. Wang et al., 2022; Xie et al., 2024). From the perspective of the relationship between hindrance stress and low motivation (Lepine et al., 2005), higher levels of hindrance stress among teachers correlate with reduced work effort, which directly contradicts the requirement of innovative work behavior for sustained individual engagement.

### 4.3. The Moderating Role of Gender

Moreover, in accordance with the proposed hypothesis, this study revealed that gender moderates the relationships between family-to-work conflict and both challenge and hindrance stress. To be specific, the relationships between family-to-work conflict and challenge stress and between family-to-work conflict and hindrance stress are stronger among men and weaker among women.

These results can be attributed to the perceptions and expectations associated with gender roles. Although men are still generally regarded as the primary financial providers for the family, they are increasingly expected to participate in domestic life, including by performing childcare and household chores. Similarly, while women are still seen as the primary caregivers within the home, their participation in the workforce has become widely accepted as a natural aspect of modern life (Cinamon & Rich, 2002). However, the significance of the work and family domains differs between male and female roles. Consequently, the impact of family-to-work conflict on work stress differs between men and women.

In line with this hypothesis, gender was observed to moderate the relationship between family-to-work conflict and challenge stress. Specifically, the relationship between family-to-work conflict and challenge stress is stronger among men but weaker among women. We believe that this finding can be understood from the following perspectives. Among men, work is typically viewed as the most important thing, and cultural expectations in Chinese society regarding men’s roles—such as those pertaining to the need to be “strong” and serve as the “pillar of the family”—make men more likely to perceive challenges, including family-to-work conflict, as a type of stress that they can and should actively overcome. In contrast, women are often expected to provide a stable home situation rather than financial security (Gutek et al., 1991). Society also often expects women to prioritize family over work in response to such conflicts. Therefore, women might not experience the same intense drive for career success as men.

In line with our expectations, gender was observed to play a moderating role in the relationship between family-to-work conflict and hindrance stress. Specifically, the relationship between family-to-work conflict and hindrance stress is stronger among men but weaker among women. We believe that this phenomenon may be attributed to the following reasons. Although men typically receive more support for their efforts to manage family–work conflicts, the fact that if family–to-work conflict is not effectively addressed, it can directly impact work performance is undeniable; in turn, this influence can negatively affect teachers’ economic income and their ability to support their families. Subsequently, this process can undermine teachers’ sense of identity and self-esteem. As a result, family-to-work conflict is likely to elicit stronger negative emotions in men than in women. In other words, men are more likely to perceive such conflicts as a form of hindrance stress. In contrast, women are less likely to face pressure to use their income to support their families. As a result, they are generally better equipped to react to and manage family-to-work conflicts in a more balanced way. Moreover, family-to-work conflict is widespread among women. Given that many women have either experienced this phenomenon personally or heard about it from others, they have largely become accustomed to this issue and have developed a certain degree of psychological preparedness in this regard. These factors contribute to the fact that women are less likely to perceive family-to-work conflict as a deeply negative or distressing source of suffering.

### 4.4. Implications and Limitations of This Research

This study has significant theoretical and practical implications. Theoretically, this study demonstrates that family-to-work conflict negatively predicts teachers’ innovative behaviors, with both challenge stress and hindrance stress serving as mediators in this relationship, while the relationships between family-to-work conflict and challenge stress and between family-to-work conflict and hindrance stress are stronger among men and weaker among women. These findings not only effectively validate the accuracy of Conservation of Resources (COR) theory and gender role theory, but also reveal that the Chinese cultural narrative of adversity containing value, combined with the high cognitive levels characteristic of university teachers, contributes to the precise predictive effects of family-to-work conflict on both challenge and hindrance stressors, which is particularly noteworthy. This represents a significant extension and refinement of the aforementioned theories.

From a practical perspective, the findings of this study offer significant guidance for enhancing teachers’ work innovation behaviors. Firstly, relevant departments should build bridges between university teachers’ families and academic institutions, helping family members understand the working conditions and characteristics of university faculty while also guiding university leaders to actively comprehend teachers’ family circumstances and needs, fostering mutual understanding and support, and reducing family-to-work conflict among educators. Secondly, managers should distinguish between stress types, effectively leveraging teachers’ challenge stressors to stimulate innovation in teaching and other work contexts, while simultaneously mitigating hindrance stressors through peer support and career counseling, and then minimize or eliminate their negative impact on innovative work behaviors. Thirdly, universities should provide gender-specific support services for teachers. For instance, offering professional training to help male teachers reduce hindrance stress, while actively challenging the traditional ‘women’s role at home’ concept for female and increasing their challenge stress by creating more career advancement opportunities, enhancing professional expectations, and actively supporting their career achievements.

Although this study reveals several meaningful findings, it also has certain limitations that highlight novel directions for future research. Firstly, this research focused solely on family-to-work conflict. However, family-to-work conflict is closely related to work-to-family conflict, and previous studies have indicated that work-to-family conflict can affect work behavior via family-to-work conflict (Song et al., 2023). Therefore, future researchers should investigate family-to-work conflict and work-to-family conflict simultaneously to produce a more comprehensive understanding of the relationship between work and family conflict and work outcomes. Secondly, in response to the call of previous studies for further refinement of the categories of work stress (Cavanaugh et al., 2000), work stress is divided into challenge stress and hindrance stress in this study. However, this distinction may still be simplistic, as previous studies have reported that challenge stress exhibits an inverted U-shaped relationship with efforts to enhance teachers’ innovative work behavior at different levels (T. Xu et al., 2024). This finding suggests that future researchers should refine the classification of work stress with the goal of exploring the mechanisms underlying the different types of work stress in more precise detail. Thirdly, the cross-sectional design of this research prevents us from drawing inferences concerning causal relationships in this context. Furthermore, some scholars have noted that the correlation between challenge stress and innovation differs significantly between studies that have featured cross-sectional designs and those that have not (J. Wang et al., 2022). Thus, longitudinal studies should be conducted to examine the relationships among these variables.

### 4.5. Conclusions

The findings of this research reveal that family-to-work conflict negatively influences innovative work behavior and that challenge stress and hindrance stress significantly mediate the relationship between family-to-work conflict and innovative work behavior. Additionally, gender significantly moderates the relationships between family-to-work conflict and challenge stress and between family-to-work conflict and hindrance stress. These results shed light on the inherent mechanisms that govern the relationship between family-to-work conflict and innovative work behavior among university teachers and highlight the significance of challenge stress, hindrance stress and gender in this context. In addition, these discoveries offer fresh insights and ideas that can be used to investigate how schools and governments can promote innovative work behavior among university teachers.

## Figures and Tables

**Table 1:** Demographic information of the participants.

Demographic Variable		Sample	
		Number	Percentage
Gender	Male	202	22.1
	Female	714	77.9
Education Background	Associate degree or below	19	2.1
	Bachelor’s degree	448	48.9
	Master’s degree or higher	449	49.0
Teaching Experience	1–3 years	156	17.0
	4–10 years	291	31.8
	11–15 years	191	20.9
	16–20 years	123	13.4
	Over 20 years	155	16.9
Professional Titles	Unrated	262	28.6
	Lecturer (Intermediate)	398	43.4
	Associate Professor (Associate Senior)	219	23.9
	Professor (Full Senior)	37	4.0

**Table 2:** Descriptive statistics and intercorrelations pertaining to the variables of interest.

	M	SD	CR	AVE	1	2	3	4
1. Family-to-work conflict	2.43	0.77	0.82	0.54	0.74	0.22	0.48	0.21
2. Challenge stress	3.76	0.75	0.92	0.66	0.20 ***	0.81	0.34	0.11
3. Hindrance stress	2.67	0.86	0.80	0.51	0.40 ***	0.30 ***	0.71	0.30
4. Innovative work behavior	4.27	0.42	0.95	0.51	−0.18 ***	0.09 **	−0.25 ***	0.71

Note: ** *p* < 0.01, *** *p* < 0.001. The data on the diagonal of questionnaires represent the square root of AVE. The data below the diagonal represent the correlation coefficients between questionnaires. The data above the diagonal represent the HTMT values of questionnaires.

**Table 3:** Multigroup analysis of differences between males and females.

Path	Gender	β	SE	95% CI	Differences in CR
FWC→CHWS	Male	0.35 ***	0.09	0.18, 0.51	2.52 ***
	Female	0.16 ***	0.05	0.07, 0.24	
FWC→HIWS	Male	0.62 ***	0.07	0.46, 0.75	2.87 **
	Female	0.37 ***	0.05	0.27, 0.46	

Note: ** *p* < 0.01, *** *p* < 0.001; FWC-family-to-work conflict; CHWS-challenge stress; HIWS-hindrance stress.

## Data Availability

The datasets generated during and analyzed during the current study are available from the corresponding author on reasonable request.
